# Coronavirus disease-19 (COVID-19) associated with acute pancreatitis: case report

**DOI:** 10.11604/pamj.2020.37.150.25873

**Published:** 2020-10-13

**Authors:** El Mehdi Simou, Mounir Louardi, Imane Khaoury, Med Amine Abidi, Akram Mansour, Aymane El Louadghiri, Kawtar Fahmaoui, Hanane Ezzouine, Boubaker Charra

**Affiliations:** 1 Department of Anesthesiology and Intensive Care, Ibn Rochd University Hospital of Casablanca, Hassan II University, Casablanca, Morocco

**Keywords:** COVID-19, SARS-CoV-2, acute pancreatitis, intensive care

## Abstract

The SARS-CoV-2 primarily attacks the respiratory system and the most common symptoms include cough, shortness of breath, and fever. However, its tropism for the digestive system has been demonstrated and its clinical digestive manifestations are increasingly recognized. Nevertheless, little attention has been paid to pancreatic lesions included in SARS-CoV-2 infection. This case describes the presentation of acute pancreatitis as a complication associated with SARS-CoV-2 infection and the importance of looking for this complication in any patient with COVID-19. Data was collected from a patient admitted with COVID-19 to intensive care in July 2020. The patient was diagnosed with acute pancreatitis associated with SARS-CoV-2. Other causes of acute pancreatitis were excluded for both patients (including alcohol, obstruction/gallstones, drugs, trauma, hypertriglyceridemia, hypercalcemia). This case highlights acute pancreatitis as a complication associated with COVID-19 and highlights the importance of measuring lipasemia and performing an abdominal computed tomography (CT) scan in patients with COVID-19.

## Introduction

A new family type of coronavirus (SARS-CoV-2) was first seen in Wuhan, China name coronavirus disease 2019 (COVID-19). It spread very quickly around the world and was accepted as a pandemic on March 11^th^, 2020. Although COVID-19 primarily attacks the respiratory system, but new clinical manifestations emerge every day and should not be neglected, even the most innocuous, and this for a better understanding of its mechanism of action and thus a better supported. Acute pancreatitis (AP) is an inflammatory disease affecting the exocrine part of the pancreatic parenchyma [1]. It is associated with high morbidity and mortality. The most common causes of acute pancreatitis are gallstones and alcohol abuse, but viral pancreatitis has been well described in the literature, mainly due to mumps, measles, coxsackia, Epstein-Barr virus and hepatitis A virus but little attention has been paid to pancreatic lesions included by SARS-CoV-2 infection.

## Patient and observation

**Figure 1:**
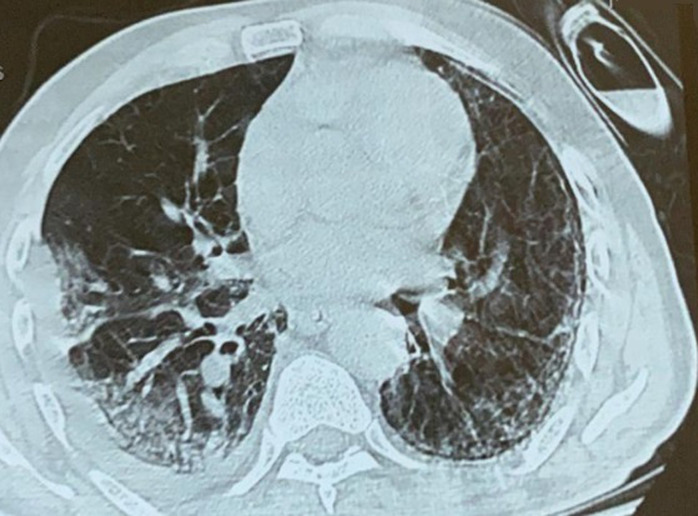
axial section of the computed tomography scan with lung parenchyma window showing a frosted glass appearance

A 67-year-old patient with a history of type 2 diabetes treated with oral antidiabetics, a cholecystectomized 10 years ago, obese with body mass index (BMI) at 34 kg/m^2^, no history of alcoholism, smoking and similar symptoms in the past. Admitted to the emergency department on July 8^th^ for febrile dyspnea, myalgia, arthralgia, without associated abdominal signs, all evolving for 10 days in a context of deterioration of the general condition. On admission, the medical examination revealed a conscious (GCS 15/15), polypneic (26 cpm) patient with an oxygen saturation (SpO2) of 85% in the ambient air and 97% when wearing a face mask without rebreather (15 L/min), blood pressure of 130/70 mmhg, pulse of 80 bpm and a temperature of 37.5°C. A chest scanner was performed which revealed typical ground glass opacities associated with crazy paving images in favor of viral pneumonia (Figure 1). The reverse transcription polymerase chain reaction (rt-PCR) for SARS-CoV-2 came back positive. The patient was transferred to medical intensive care unit dedicated to COVID-19.

**The biological assessment:** the complete blood count showed white blood cells at 5940/µL, neutrophils at 4650/ μL, lymphocyte at 910/μL, hemoglobin at 14 g/dl, platelets at 161,000/ μL. Fibrinogen at 7.27 g/L, CRP at 41.3 mg/L, PCT at 0.06 ng/ml, ferritin at 1079.5 ng/L, calcemia at 1.94mmol/L, creatinine at 6.55 mg/L, urea at 3.3 mmol/l, ALT at 33UI/L, AST at 26UI/L, ALP at 80 UI/L, GGT at 35 UI/L, triglycerides at 2.40 mmol/L and troponins at 8.1 ng/L. The electrocardiogram showed regular sinus rhythm of 80 bpm, QT at 420 mm without repolarization disorders. The echocardiography did not show any abnormalities. Undilated ventricular chambers with a systolic ejection fraction at 60%, no valve abnormality, and no signs of pulmonary hypertension or acute cor pulmonale.

**Figure 2:**
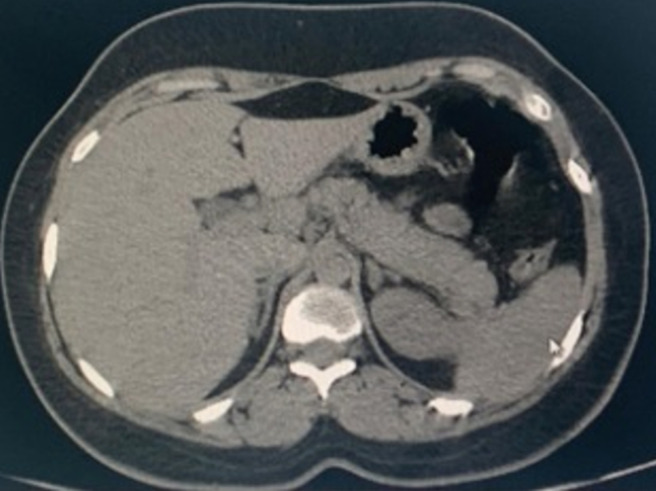
abdominal CT scan in axial section showing a tumefied pancreas with loss of its physiological lobulations associated with an infiltration of peripancreatic fat

Therapeutic management included, oxygen therapy, the association hydroxychloroquine 200 mg three times per day added to azithromycin 500 mg the first day then 250 mg per day and methylprednisolone at 80 mg per day for 7 days, vitamin C 1 gramme per day and zinc 90 mg per day and low molecular weight heparin enoxaparin 100 UI/kg/day. On day 5, the patient was placed on assisted ventilation. Faced with the persistence of a fever estimated at 40 degrees Celsius, an infectious assessment including a cytobacteriological examination of the urine, a protected distal bronchial sample, blood cultures, a lumbar puncture and an abdominal and pelvic ultrasound were requested and not objectifying no anomaly. On the 13^th^ day, a thoraco-abdomino-pelvic CT was performed objectifying an acute stage c pancreatitis according to the balthazar classification (Figure 2). The lipasemia was at 576 UI/L. The serologies (HAV, VHB, VHC, VHD, VHE, HSV, VZV, EBV, CMV, HIV) were carried out and which were negative. The patient was put to digestive rest and received antibiotics. However, the patient had an unfavorable outcome and died on day 18.

## Discussion

Acute pancreatitis is an inflammatory disease affecting the exocrine part of the pancreatic parenchyma [1]. Gallstones and alcoholism are the most common etiologies of acute pancreatitis. Cholelithiasis is reported in 40 to 70% while alcoholism is found in 25 to 35% of cases. Drug-induced pancreatitis accounts for 1-2% of all pancreatitis. There are several known pancreatotoxic drugs including immunosuppressive drugs, allupirunol, furosemide, tricyclic antibiotics, metronidazole and others. It is defined by the occurrence of pancreatitis shortly after the introduction of a drug or after increasing its doses, and this in the absence of a classic cause of pancreatitis (in order of frequency under our skies: cholelithiasis, metabolic and alcoholic) [2]. The metabolic origin is possible: (hypercalcemia and hypertriglyceridemia), all causes of hypercalcemia can be responsible for acute pancreatitis. However, in the largest series of hyperparathyroidism (1153 cases), the frequency of acute pancreatitis is only 1.5% of patients, making hypercalcemia a rare cause of acute pancreatitis [3], and this suggests to us that other factors are necessary for the development of acute pancreatitis. For hypertriglyceridemia, serum triglycerides must exceed 1000 mg/dL to be considered a possible cause of acute pancreatitis. Benign or malignant strictures of the pancreatic duct are associated with acute pancreatitis in about 5-14% of patients. And about 10% of cases of acute pancreatitis are caused by other factors such as infection with parasites, bacteria, and virus [4]. Viruses are the agents most frequently implicated in the development of acute infectious pancreatitis, and these include (hepatotropic virus, coxsackie virus, cytomegalovirus (CMV), human immunodeficiency virus (HIV), and other viruses [5]. However, additional investigation should be performed to rule out other etiologies of acute infectious pancreatitis and to correlate the infectious agent with the occurrence of this disease and this for better patient management.

During the 2002-2004 epidemic, the SARS-CoV virus bound to the Angiotensin-converting enzyme 2 (ACE2) receptor. Harmer and his colleagues have demonstrated the expression of ACE2 mRNA in several tissues, including pancreatic tissue [6]. Furthermore, the genomic sequences showed that SARS-CoV-2 shared 79.6% sequence identity with SARS-CoV, both encoding and expressing the spike (S) glycoproteins that could bind to the receptor ACE2 to enter human cells [7]. Thus, the presence of ACE2 in the pancreas indicates the latter as a potential target for COVID-19 and consequently the possibility of developing acute pancreatitis during SARS-CoV-2 infection. Two studies carried out, one in the United States and the other in Denmark, have reported cases of acute pancreatitis associated with COVID-19 [8, 9]. In our case, the absence of other obvious etiologies explaining the occurrence of acute pancreatitis, the timing of onset of acute pancreatitis versus onset of symptoms and lack of perfect knowledge of the possible clinical manifestations of COVID-19, it is assumed that the acute pancreatitis is due to infection by the coronavirus.

The diagnosis of acute pancreatitis consists of the presence of two of these three signs: abdominal pain, amylase or lipase > 3 times the upper normal limit and characteristic finds on diagnostic imaging. Previous studies have reported the association of acute pancreatitis and COVID-19, all of these reported cases presented with abdominal symptomatology associated with the respiratory signs which led to the realization of a biological assessment made of lipasemia and amylasemia and whose values were increased thus requiring to complete the assessment by abdominal ultrasound or an abdominal scanner for the confirmation of acute pancreatitis [8, 9]. However, in our case the abdominal signs were absent and the symptomatology was mainly respiratory. Acute pancreatitis is a serious disease with high morbidity and mortality, it can be complicated by multiple organ failure, including respiratory distress. COVID-19 may also be the cause of multiple organ failure and therefore it cannot be concluded that acute pancreatitis contributed to our patient's fatal outcome. In addition, this prompts us to suggest measuring lipasemia and performing an abdominal CT scan to detect acute pancreatitis even in the absence of clinical signs in any patient with COVID-19.

## Conclusion

Acute pancreatitis is a disease of varying severity and its association with COVID-19 can be fatal. This reported case underscored the importance of assaying lipasemia and performing an abdominal CT scan in any patient with COVID-19.
